# Establishment of prognostic nomogram for elderly colorectal cancer patients: a SEER database analysis

**DOI:** 10.1186/s12876-020-01464-z

**Published:** 2020-10-20

**Authors:** Chaoran Yu, Yujie Zhang

**Affiliations:** 1 Fudan University Shanghai Cancer Center, Fudan University, Dongan Road 270, Shanghai, 200025 P. R. China; 2 Department of Oncology, Shanghai Medical College, Fudan University, Dongan Road 270, Shanghai, 200025 P. R. China; 3 Department of Gastrointestinal Surgery, Tongji Hospital, Tongji Medical College in Huazhong University of Science and Technology, Wuhan, Hubei China

**Keywords:** Elderly colon cancer, Nomogram, Overall survival, Cancer-specific survival, SEER

## Abstract

**Background:**

This study aimed to establish nomogram models of overall survival (OS) and cancer-specific survival (CSS) in elderly colorectal cancer (ECRC) patients (Age ≥ 70).

**Methods:**

The clinical variables of patients confirmed as ECRC between 2004 and 2016 were retrieved from the Surveillance, Epidemiology, and End Results (SEER) database. Univariate and multivariate analysis were performed, followed by the construction of nomograms in OS and CSS.

**Results:**

A total of 44,761 cases were finally included in this study. Both C-index and calibration plots indicated noticeable performance of newly established nomograms. Moreover, nomograms also showed higher outcomes of decision curve analysis (DCA) and the area under the curve (AUC) compared to American Joint Committee on Cancer (AJCC) tumor-node-metastasis (TNM) stage and SEER stage.

**Conclusions:**

This study established nomograms of elderly colorectal cancer patients with distinct clinical values compared to AJCC TNM and SEER stages regarding both OS and CSS.

## Background

Colorectal cancer has been ranked as the second most common malignancy in women and third in men across the world. Annual global incidence is approximately 1.4 million with nearly 700,000 deaths [1, 2]. There are more than 50,000 death reports and over 130,000 newly occurred cases in the United States [2]. In European Union, 215,000 cases have been reported with colorectal cancer being listed as the second common cause of death [3]. In China, colorectal cancer is listed as one of the five most commonly malignancies both in men and women [4].

Genomic characterization of colorectal cancer has been well elucidated and the role of immunology is increasingly valued [5–7]. Therapeutically, surgical intervention and chemotherapy-based strategies have been widely accepted for colorectal cancer [8, 9]. Noteworthy, the impact of colorectal cancer surgery on the elder group, regarding long term survival, is similar to that of younger group [10].

Generally, elderly colorectal cancer patients (ECRC), defined by age surpass 70 years old, may naturally associate with increased mortality as age increased. However, no study did fully cover nor depict the quantified association of age and risks for prognosis of ECRC [11, 12]. Previously, tumor-node-metastasis (TNM) stage system of American Joint Committee on Cancer (AJCC) is widely used in the therapeutic and prognostic administration of colorectal cancer. Given increasing values of multiple variables, including tumor size and marital status, have been noticed [13, 14], a more comprehensive prognostic predictor is necessary for ECRC.

Of note, knowledge regarding the clinical prediction of ECRC is limited, with very few studies focusing on the nomogram implementation. In this study, a ECRC-targeting nomogram was established for prognostic prediction based on large sample size retrieved from the Surveillance, Epidemiology, and End Results (SEER) database in hopes of elucidating further prognostic insights [15].

## Methods

### Recruitment of patients from SEER database

The clinical variables of patients confirmed as ECRC between 2004 and 2016 were retrieved from the SEER database, a program established by National Cancer Institute aiming for comprehensively national-level clinical investigation [16, 17]. The reference number was 16,595-Nov2018. The inclusion criteria were: 1) colon and rectum (site recode, international classification of diseases for oncology (ICD-O-3)/WHO 2009); 2) age ≥ 70; 3) complete information on TNM stage; 4) only one primary tumor cases were selected; 5) surgery performed in each case. Next, all included cases were randomly divided into training and validation sets with equal sample size. In addition, x-tile software was used to determine and visualize the best cutoff points of age and tumor size variables in this study [18].

### Clinical variables extracted for analysis

Age, sex, marital status, tumor site, histological grade, SEER stage, the AJCC TNM stage, distant metastasis (bone, brain, liver and lung) and tumor size were all selected for the establishment of nomogram modeling. Regarding the clinical outcome, overall survival (OS) and cancer-specific survival (CSS) were chosen as the primary and second endpoints.

### Construction and validation of the nomogram

Statistically, chi-square test was used for all included categories between training and validation groups. Next, univariate and multivariate analysis were used to determine distinct variables, which were further output for the construction of nomogram model by R software 3.3.0 (R Foundation for Statistical Computing, Vienna, Austria, www.r-project.org). Then, the validation group was used for the assessment of the newly established nomogram. The comparison between the nomogram prediction and observed outcomes was assessed by the concordance index (C-index). The calibration plot was used for visualized comparison between prognosis predicted by nomogram and actual ones. Sensitivity and specificity were evaluated by receiver operating characteristics curve (ROC)-the area under the curve (AUC). Furthermore, the power of nomogram model was also compared to the TNM stage and SEER stage in both ROC and decision curve analysis (DCA). All analysis was achieved by R software 3.3.0, with *p* value< 0.05 considered as statistically significant.

## Results

### Characterization of included cases

**Fig. 1:**
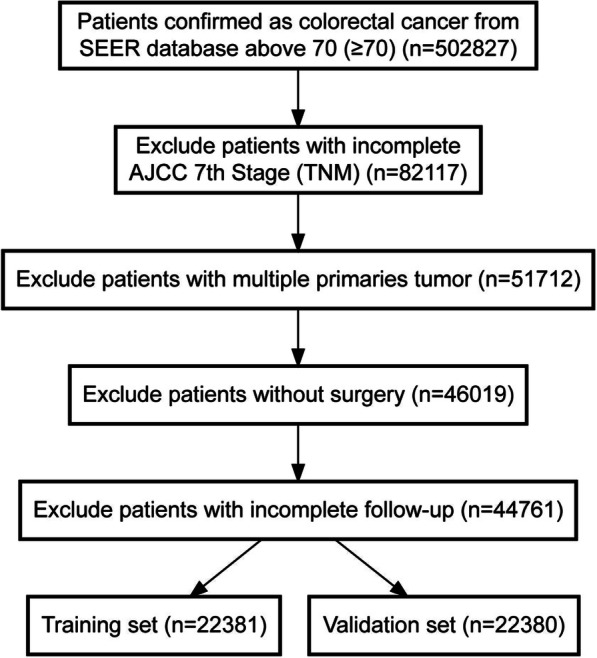
The inclusion criteria flowchart of recruited patients in SEER database

**Fig. 2:**
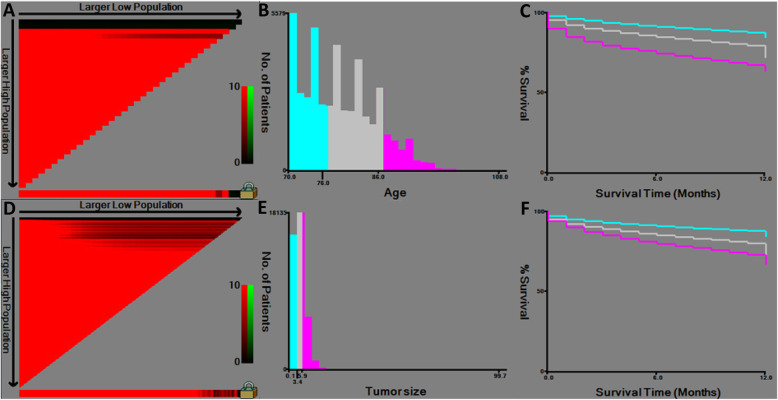
The X-tile analysis of best-cutoff points of age and tumor size variables. **a** X-tile plot of training sets in age; **b** the cutoff point was highlighted using a histogram of the entire cohort; **c** the distinct prognosis determined by the cutoff point was shown using a Kaplan-Meier plot (low subset = blue, middle subset = gray, high subset = magenta); **d** X-tile plot of training sets in tumor size; **e** the cutoff point was highlighted using a histogram; **f** Kaplan-Meier plot of prognosis determined by the cutoff point (low subset = blue, middle subset = gray, high subset = magenta)

Following inclusion criteria, a total of 44,761 cases were finally included in this study with 22,381 assigned to training set and 22,380 to validation set randomly (Fig. 1). Among all patients, 44.6% were male and 55.4% female; 47.6% were unmarried and 46.8% married; 81.9% were colon cancer and 18.1% rectal cancer; 0.3% of cases had bone metastasis, 0.1% with brain metastasis, 7.0% with liver metastasis, 1.8% with lung metastasis. The cutoff points of age and tumor size were determined by x-tile (Fig. 2). Specifically, 40.9% were < =76 years old, 44.5% between 77 and 86 years old, and 14.7% > =87 years old. 29.8% were < =3.4 cm, 36.3% between 3.5–5.9 cm and 25.4% > = 6 cm (Table 1). No significant difference was identified between training and validation cohorts regarding each included variable.

**Table 1:** Baseline demographic and clinical characteristics of elderly patients with CRC

Variables	Total (***n*** = 44,761)	Training cohort (***n*** = 22,381)	Validation cohort (***n*** = 22,380)	***P***^***#***^
**Sex**				0.105
Male	19,969 (44.6)	10,070 (45.0)	9899 (44.2)	
Female	24,792 (55.4)	12,311 (55.0)	12,481 (55.8)	
**Age**				0.953
< =76	18,287 (40.9)	9148 (40.9)	9139 (40.8)	
77–86	19,901 (44.5)	9937 (44.4)	9964 (44.5)	
> =87	6573 (14.7)	3296 (14.7)	3277 (14.6)	
**Marital status**				0.310
Unmarried	21,287 (47.6)	10,563 (47.2)	10,724 (47.9)	
Married	20,927 (46.8)	10,534 (47.1)	10,393 (46.4)	
Unknown	2547 (5.7)	1284 (5.7)	1263 (5.6)	
**Tumor site**				0.530
Colon	36,652 (81.9)	18,352 (82.0)	18,300 (81.8)	
Rectum	8109 (18.1)	4029 (18.0)	4080 (18.2)	
**Grade**				0.346
I	3870 (8.6)	1919 (8.6)	1951 (8.7)	
II	29,426 (65.7)	14,665 (65.5)	14,761 (66.0)	
III	7478 (16.7)	3781 (16.9)	3697 (16.5)	
IV	1642 (3.7)	806 (3.6)	836 (3.7)	
Unknown	2345 (5.2)	1210 (5.4)	1135 (5.1)	
**SEER_stage**				0.994
Localized	19,923 (44.5)	9957 (44.5)	9966 (44.5)	
Regional	19,512 (43.6)	9758 (43.6)	9754 (43.6)	
Distant	5326 (11.9)	2666 (11.9)	2660 (11.9)	
**AJCC_stage**				0.797
I	12,173 (27.2)	6065 (27.1)	6108 (27.3)	
II	14,656 (32.7)	7300 (32.6)	7356 (32.9)	
III	13,071 (29.2)	6581 (29.4)	6490 (29.0)	
IV	4861 (10.9)	2435 (10.9)	2426 (10.8)	
**AJCC_T**				0.674
T1	7352 (16.4)	3665 (16.4)	3687 (16.5)	
T2	6570 (14.7)	3243 (14.5)	3327 (14.9)	
T3	23,269 (52.0)	11,669 (52.1)	11,600 (51.8)	
T4	7570 (16.9)	3804 (17.0)	3766 (16.8)	
**AJCC_N**				0.271
N0	27,879 (62.3)	13,893 (62.1)	13,986 (62.5)	
N1	10,677 (23.9)	5410 (24.2)	5267 (23.5)	
N2	6205 (13.9)	3078 (13.8)	3127 (14.0)	
**AJCC_M**				0.893
M0	39,900 (89.1)	19,946 (89.1)	19,954 (89.2)	
M1	4861 (10.9)	2435 (10.9)	2426 (10.8)	
**Bone metastasis**				0.990
No	44,314 (99.0)	22,158 (99.0)	22,156 (99.0)	
Yes	119 (0.3)	60 (0.3)	59 (0.3)	
Unknown	328 (0.7)	163 (0.7)	165 (0.7)	
**Brain metastasis**				0.700
No	44,375 (99.1)	22,193 (99.2)	22,182 (99.1)	
Yes	41 (0.1)	22 (0.1)	19 (0.1)	
Unknown	345 (0.8)	166 (0.7)	179 (0.8)	
**Liver metastasis**				0.978
No	41,350 (92.4)	20,677 (92.4)	20,673 (92.4)	
Yes	3138 (7.0)	1566 (7.0)	1572 (7.0)	
Unknown	273 (0.6)	138 (0.6)	135 (0.6)	
**Lung metastasis**				0.586
No	43,655 (97.5)	21,837 (97.6)	21,818 (97.5)	
Yes	784 (1.8)	379 (1.7)	405 (1.8)	
Unknown	322 (0.7)	165 (0.7)	157 (0.7)	
**Tumor size**				0.678
< =3.4	13,341 (29.8)	6625 (29.6)	6716 (30.0)	
3.5–5.9	16,250 (36.3)	8142 (36.4)	8108 (36.2)	
> =6	11,387 (25.4)	5736 (25.6)	5651 (25.3)	
Unknown	3783 (8.5)	1878 (8.4)	1905 (8.5)	

^#^ Chi-square test

### Establishment of the nomogram

**Fig. 3:**
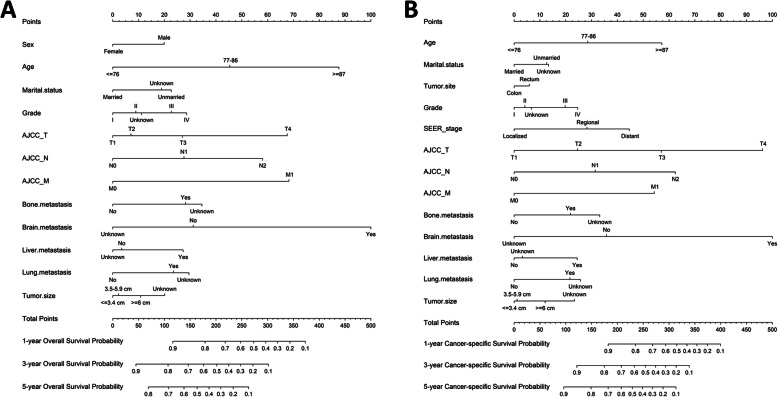
Establishment of overall survival (OS) and cancer-specific survival (CSS) nomograms. **a** Construction of OS nomogram; **b** construction of CSS nomogram

Interestingly, sex, age, marital status, tumor size, grade, SEER stage, AJCC TNM stage, bone metastasis, brain metastasis, liver metastasis, lung metastasis and tumor size were all displayed high statistically difference in univariate OS analysis (Table 2). Next, sex, age, marital status, grade, AJCC TNM, bone metastasis, brain metastasis, liver metastasis and lung metastasis and tumor size were all significantly identified in OS multivariate analysis (Table 2). Meanwhile in CSS, age, marital status, tumor site, grade, SEER stage, AJCC TNM stage, bone metastasis, brain metastasis, liver metastasis, lung metastasis and tumor size were significantly identified in univariate CSS analysis. Age, marital status, tumor site, grade, SEER stage, AJCC TNM, bone metastasis, brain metastasis, liver metastasis, lung metastasis and tumor size were significantly associated with CSS in multivariate analysis (Table 3). Thus, OS and CSS nomogram models of 1-, 3- and 5-year were established, respectively (Fig. 3a, b).

**Table 2:** Univariate and multivariate analysis of overall survival in the training cohort

Variables	Univariate analysis	Multivariate analysis	
	***P***	HR (95% CI)	***P***
**Sex**	0.060		
Male		Reference	
Female		0.786(0.752–0.822)	< 0.001
**Age**	< 0.001		
< =76		Reference	
77–86		1.725(1.643–1.811)	< 0.001
> =87		2.868(2.699–3.047)	< 0.001
**Marital status**	< 0.001		
Unmarried		Reference	
Married		0.762(0.727–0.798)	< 0.001
Unknown		0.957(0.873–1.050)	0.351
**Tumor site**	< 0.001		
Colon		Reference	
Rectum		0.991(0.936–1.050)	0.765
**Grade**	< 0.001		
I		Reference	
II		1.114(1.022–1.215)	0.014
III		1.315(1.195–1.447)	< 0.001
IV		1.413(1.247–1.601)	< 0.001
Unknown		1.146(1.005–1.307)	0.042
**SEER_stage**	< 0.001		
Localized		Reference	
Regional		1.047(0.973–1.126)	0.222
Distant		1.181(0.975–1.431)	0.088
**AJCC_stage**	< 0.001		
I		–	–
II		–	–
III		–	–
IV		–	–
**AJCC_T**	< 0.001		
T1		Reference	
T2		1.083(0.979–1.199)	0.123
T3		1.353(1.233–1.486)	< 0.001
T4		2.173(1.953–2.418)	< 0.001
**AJCC_N**	< 0.001		
N0		Reference	
N1		1.365(1.282–1.453)	< 0.001
N2		1.975(1.845–2.113)	< 0.001
**AJCC_M**	< 0.001		
M0		Reference	
M1		2.017(1.662–2.448)	< 0.001
**Bone metastasis**	< 0.001		
No		Reference	
Yes		1.393(1.058–1.835)	0.018
Unknown		1.507(0.909–2.500)	0.112
**Brain metastasis**	< 0.001		
No		Reference	
Yes		2.145(1.401–3.285)	< 0.001
Unknown		0.687(0.415–1.135)	0.142
**Liver metastasis**	< 0.001		
No		Reference	
Yes		1.329(1.209–1.462)	< 0.001
Unknown		0.962(0.684–1.352)	0.822
**Lung metastasis**	< 0.001		
No		Reference	
Yes		1.327(1.178–1.495)	< 0.001
Unknown		1.432(1.033–1.984)	0.031
**Tumor size**	< 0.001		
< =3.4		Reference	
3.5–5.9		1.026(0.968–1.088)	0.379
> =6		1.137(1.069–1.210)	< 0.001
Unknown		1.272(1.156–1.398)	< 0.001

**Table 3:** Univariate and multivariate analysis of cancer-specific survival in the training cohort

Variables	Univariate analysis	Multivariate analysis	***P***
	***P***	HR (95% CI)	
**Sex**	0.644		
Male		–	–
Female		–	–
**Age**	< 0.001		
< =76		Reference	
77–86		1.499(1.412–1.592)	< 0.001
> =87		2.252(2.083–2.435)	< 0.001
**Marital status**	< 0.001		
Unmarried		Reference	
Married		0.836(0.791–0.884)	< 0.001
Unknown		1.011(0.896–1.140)	0.865
**Tumor site**	< 0.001		
Colon		Reference	
Rectum		1.088(1.011–1.171)	0.024
**Grade**	< 0.001		
I		Reference	
II		1.061(0.943–1.194)	0.326
III		1.324(1.168–1.502)	< 0.001
IV		1.417(1.212–1.657)	< 0.001
Unknown		1.100(0.916–1.321)	0.308
**SEER_stage**	< 0.001		
Localized		Reference	
Regional		1.492(1.343–1.657)	< 0.001
Distant		1.883(1.507–2.354)	< 0.001
**AJCC_stage**	< 0.001		
I		–	–
II		–	–
III		–	–
IV		–	–
**AJCC_T**	< 0.001		
T1		Reference	
T2		1.417(1.185–1.694)	< 0.001
T3		2.244(1.912–2.634)	< 0.001
T4		3.914(3.301–4.640)	< 0.001
**AJCC_N**	< 0.001		
N0		Reference	
N1		1.561(1.444–1.687)	< 0.001
N2		2.426(2.237–2.631)	< 0.001
**AJCC_M**	< 0.001		
M0		Reference	
M1		2.160(1.743–2.677)	< 0.001
**Bone metastasis**	< 0.001		
No		Reference	
Yes		1.360(1.021–1.812)	0.036
Unknown		1.600(0.934–2.743)	0.087
**Brain metastasis**	< 0.001		
No		Reference	
Yes		2.424(1.564–3.756)	< 0.001
Unknown		0.602(0.346–1.049)	0.073
**Liver metastasis**	< 0.001		
No		Reference	
Yes		1.414(1.280–1.563)	< 0.001
Unknown		1.047(0.724–1.514)	0.807
**Lung metastasis**	< 0.001		
No		Reference	
Yes		1.359(1.200–1.539)	< 0.001
Unknown		1.439(1.020–2.029)	0.038
**Tumor size**	< 0.001		
< =3.4		Reference	
3.5–5.9		1.017(0.942–1.097)	0.670
> =6		1.186(1.095–1.283)	< 0.001
Unknown		1.394(1.221–1.592)	< 0.001

### Nomogram validation

**Fig. 4:**
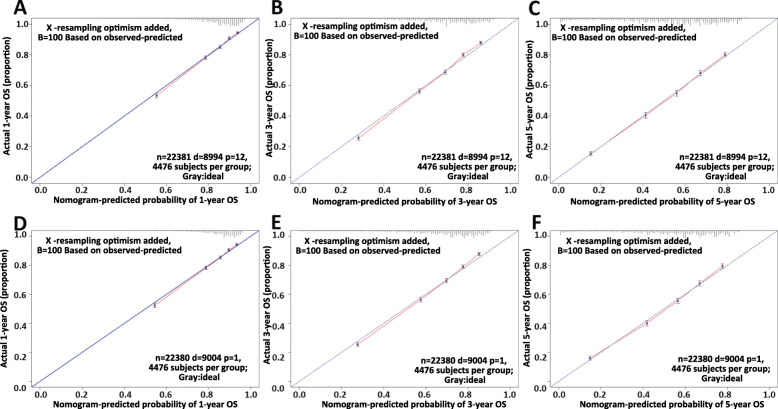
Calibration plots of OS nomogram model. **a** 1-year calibration plot of OS using training set; **b** 3-year calibration plot of OS using training set; **c** 5-year calibration plot of OS using training set; **d** 1-year calibration plot of OS using validation set; **e** 3-year calibration plot of OS using validation set; **f** 5-year calibration plot of OS using validation set

**Fig. 5:**
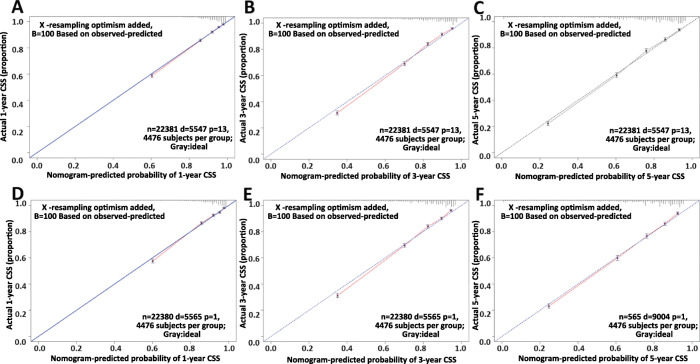
Calibration plots of CSS nomogram model. **a** 1-year calibration plot of CSS using training set; **b** 3-year calibration plot of CSS using training set; **c** 5-year calibration plot of CSS using training set; **d** 1-year calibration plot of CSS using validation set; **e** 3-year calibration plot of CSS using validation set; **f** 5-year calibration plot of CSS using validation set

**Fig. 6:**
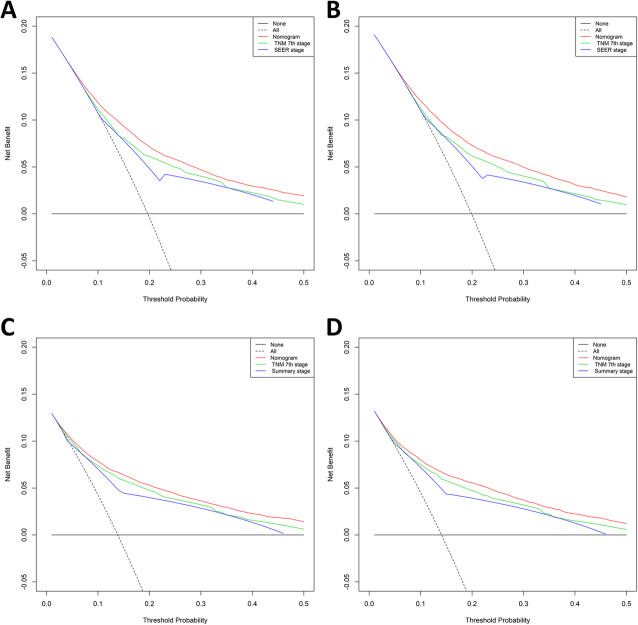
Decision curve analysis (DCA) of OS and CSS nomograms. **a** DCA of OS nomogram using training set; **b** DCA of OS nomogram using validation set; **c** DCA of CSS nomogram using training set; **d** DCA of CSS nomogram using validation set

**Fig. 7:**
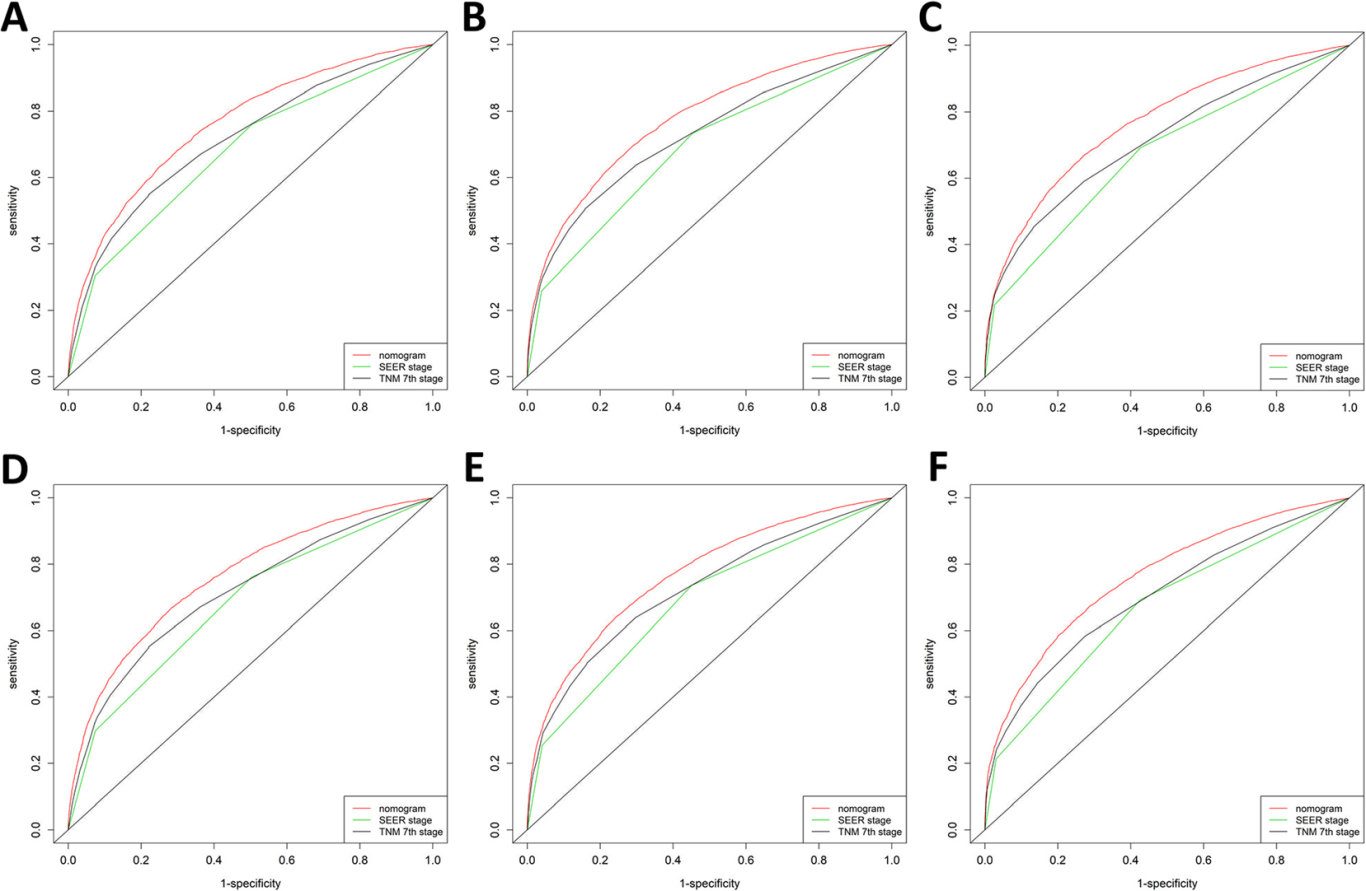
Receiver operating characteristics curve (ROC) comparison of OS nomogram, AJCC TNM stage and SEER stage. **a**1-year ROC of OS nomogram using train set; **b** 3-year ROC of OS nomogram using training set; **c** 5-year ROC of OS nomogram using training set; **d** 1-year ROC of OS nomogram using validation set; **e** 3-year ROC of OS nomogram using validation set; **f** 5-year ROC of OS nomogram using validation set

**Fig. 8:**
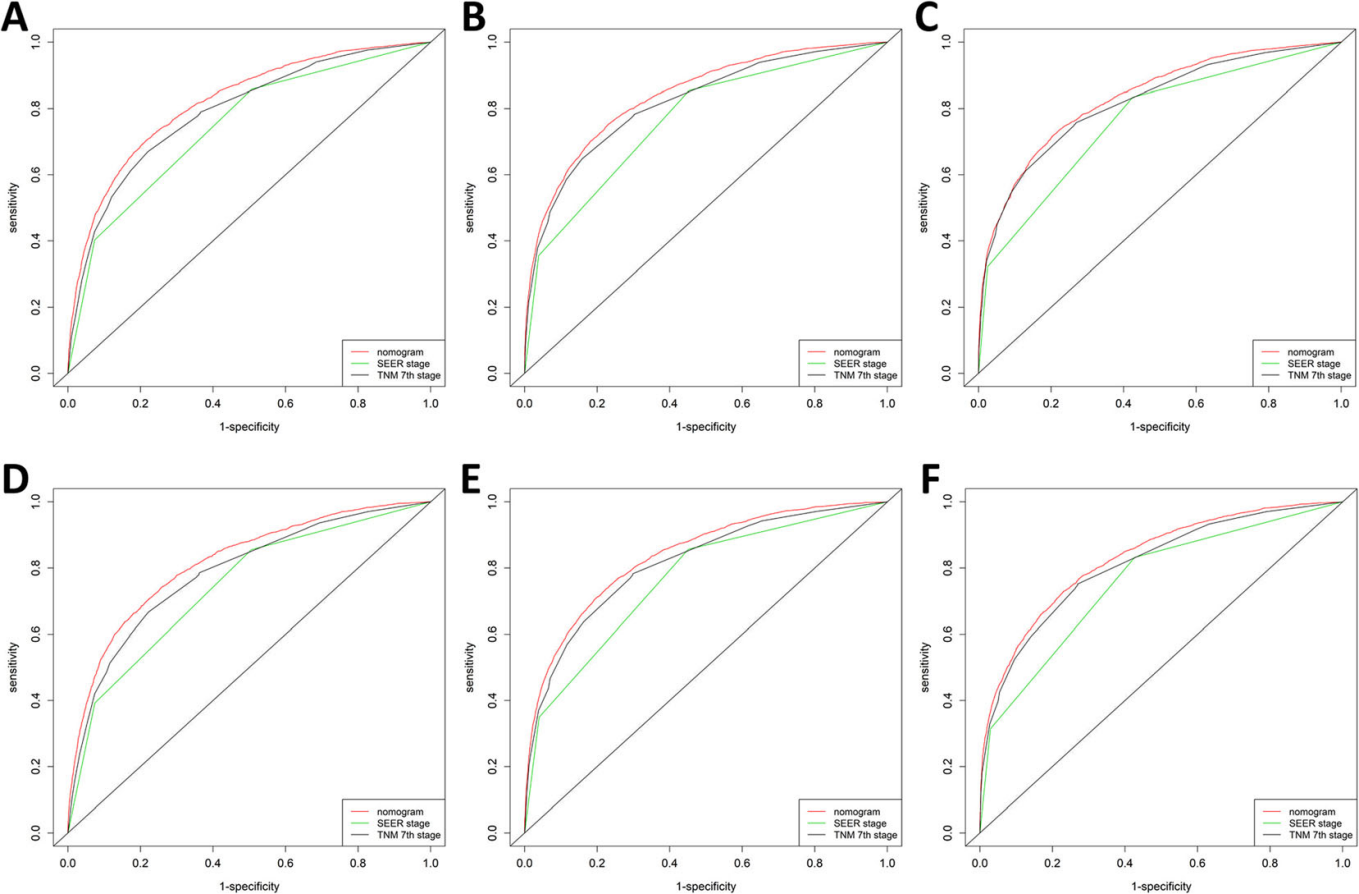
ROC comparison of CSS nomogram, AJCC TNM stage and SEER stage. **a** 1-year ROC of CSS nomogram using train set; **b** 3-year ROC of CSS nomogram using training set; **c** 5-year ROC of CSS nomogram using training set; **d** 1-year ROC of CSS nomogram using validation set; **e** 3-year ROC of CSS nomogram using validation set; **f** 5-year ROC of CSS nomogram using validation set

The assessment was performed both internally and externally, measured by C-index and calibration plots. Specifically, C-index of OS nomogram was 0.726 (95% confidence interval (95%CI): 0.720–0.732) in training set while 0.722 (95%CI: 0.716–0.728) in validation set (Table 4. C-index of CSS was 0.791 (95%CI: 0.785–0.797) in training set while 0.789 (95%CI: 0.783–0.795) (Table 4). Meanwhile, calibration plots indicated high quality of predicted outcome of OS/CSS nomogram models (Figs. 4, 5). Next, to further compare the nomograms with other classic staging methods, including AJCC TNM stage and SEER stage, DCA and ROC were performed in both OS and CSS. In DCA, nomograms both in OS and CSS showed superior power to AJCC TNM stage and SEER stage (Fig. 6). Meanwhile, nomograms in OS and CSS also showed higher statistic power to AJCC TNM stage and SEER stage (Figs. 7, 8, Table 5).

**Table 4:** C-indexes for the nomograms and other stage systems in patients with CRC

Survival		Training set			Validation set		
		HR	95%CI	*P*	HR	95%CI	*P*
OS	Nomogram	0.726	0.720–0.732	Reference	0.722	0.716–0.728	Reference
	SEER stage	0.649	0.643–0.655	< 0.001	0.65	0.644–0.656	< 0.001
	7th edition TNM stage	0.682	0.676–0.688	< 0.001	0.681	0.675–0.687	< 0.001
CSS	Nomogram	0.791	0.785–0.797	Reference	0.789	0.783–0.795	Reference
	SEER stage	0.728	0.721–0.735	< 0.001	0.727	0.720–0.734	< 0.001
	7th edition TNM stage	0.77	0.763–0.777	< 0.001	0.767	0.760–0.774	< 0.001

**Table 5:** The area under the curve (AUC) of comparison between nomograms and AJCC TNM stage and the Surveillance, Epidemiology, and End Results (SEER) database stage

Survival		AUC					
		Training set			Validation set		
		1-year	3-year	5-year	1-year	3-year	5-year
OS	Nomogram	0.760	0.774	0.766	0.758	0.768	0.760
	SEER stage	0.677	0.685	0.670	0.676	0.685	0.668
	7th edition TNM stage	0.714	0.725	0.712	0.710	0.723	0.702
CSS	Nomogram	0.819	0.839	0.833	0.817	0.837	0.830
	SEER stage	0.746	0.764	0.763	0.742	0.764	0.758
	7th edition TNM stage	0.790	0.817	0.817	0.785	0.814	0.810

## Discussion

Up to now, numerous studies had investigated the role of prognostic nomograms for colorectal cancer patients using SEER database for variable objects [19, 20]. In fact, increasing studies tended to focus more on the therapeutics or modified classification, with very rare highlighted the role of age in the prognostic assessment of colorectal cancer. Our previous study reported that a nomogram for early-onset colorectal cancer patients could display comparably higher C-index value and better performance than conventional variables [21]. ECRC, on the other hand, had been explored with limited studies. Li et al. reported that, with 18,937 included cases, adjuvant chemotherapy did not offer additional survival benefits to elderly patients with stage II or III [22]. Nonetheless, a general prognostic nomogram of ECRC is yet to be fully characterized. In this study, the nomograms displayed higher C-index and convinced calibration plots for OS and CSS prediction using SEER database. Moreover, they achieved higher values regarding both AUC and DCA assessment systems compared to AJCC TNM and SEER stages.

Of note, in OS, 12 variables (sex, age, marital status, grade, AJCC TNM, bone metastasis, brain metastasis, liver metastasis and lung metastasis and tumor size) out of 15 variables were determined for the construction of nomogram. Similar feature had also been noticed in CSS nomogram. It was highly possible that the prognosis of ECRC could be associated with more variables than common colorectal cancer cases. Moreover, four types of distant metastasis, for the first time, had been incorporated for nomogram of ECRC in SEER analysis.

In addition, X-tile tool was introduced for the best cutoff values of age and tumor size in this study. X-tile tool was established as a powerful graphic method to illustrate potential subsets (cutoff) with construction of a two dimensional projection [18]. It had been widely used in numerous investigations, including esophageal squamous cell carcinoma, bladder cancer and chondrosarcoma [23–25]. In this study, for the first time, subsets of consecutive variables, age and tumor size, were determined by X-tile tool. In fact, the role of tumor size had been intensively studied [26]. However, the cutoff points of tumor size in colorectal cancer remain largely arbitrary. Therefore, introduction of X-tile for the classification of tumor size could be both reliable and replicated.

Generally, elderly patients may naturally associate with increased mortality as age increased. However, no study did fully cover nor depict the quantified association of age and risks for prognosis, particularly when elderly patients had surpassed 70 years old. In our study, age itself was identified as a higher risk factor in OS compared to CSS nomogram, with age ≥ 87 representing nearly 90 points in OS but less than 60 points in CSS. Interestingly, female was identified as a protective factor in OS nomogram, instead of CSS nomogram. Moreover, marriage is also identified as a protective factor in both OS and CSS nomogram. By comparing OS and CSS nomograms, insightful clues had been noticed for further external clinical investigation.

## Conclusion

This study established nomograms of elderly colorectal cancer patients with distinct clinical values compared to AJCC TNM and SEER stages regarding both OS and CSS.

## Data Availability

The data that support the findings of this study are available from SEER database but restrictions apply to the availability of these data, which were used under license for the current study (ID: 16595-Nov2018), and so are not publicly available. Data are however available from the authors upon reasonable request and with permission of SEER database.

## Abbreviations

HR: Hazard ratio
95%CI: 95% confidence intervals
OS: Overall survival
CSS: Cancer-specific survival
ECRC: Elderly colorectal cancer
SEER: Surveillance, Epidemiology, and End Results database
TNM: Tumor-Node-Metastasis stage
AJCC: American Joint Committee on Cancer
C-index: Concordance index
ROC: Receiver operating characteristics curve
DCA: Decision curve analysis
AUC: Area under receiver operating characteristics curve
